# Comparing physicians’ and patients’ reporting on adverse reactions in randomized trials on acupuncture—a secondary data analysis

**DOI:** 10.1186/s12906-019-2638-x

**Published:** 2019-08-22

**Authors:** Thea Schwaneberg, Claudia M. Witt, Stephanie Roll, Daniel Pach

**Affiliations:** 1 Department of Vascular Medicine, Working Group GermanVasc, University Medical Center Hamburg-Eppendorf, Hamburg, Germany; 2 Charité – Universitätsmedizin Berlin, corporate member of Freie Universität Berlin, Humboldt-Universität zu Berlin, and Berlin Institute of Health, Institute for Social Medicine, Epidemiology and Health Economics, Berlin, Germany; 3 Institute of Complementary and Integrative Medicine, University Hospital Zurich and University of Zurich, Zurich, Switzerland

**Keywords:** Acupuncture, Adverse reaction, Safety documentation, Patients’ reports

## Abstract

**Background:**

We aimed to compare patients’ and physicians’ safety reporting using data from large acupuncture trials (44,818 patients) and to determine associations between patient characteristics and reporting of adverse reactions.

**Methods:**

Six pragmatic randomized trials with an additional non-randomized study arm that included those patients who refused randomization were evaluated. Patients received acupuncture treatment for osteoarthritis of the hip or knee, chronic neck pain, chronic low back pain, chronic headache, dysmenorrhea, or allergic rhinitis or asthma. Safety outcomes were evaluated by questionnaires from both the physicians and the patients. To determine level of agreement between physicians and patients on the prevalence of adverse reactions, Cohen’s kappa was used. With multilevel models associations between patient characteristics and reporting of adverse reactions were assessed.

**Results:**

Patients reported on average three times more adverse reactions than the study physicians: for bleeding/haematoma, 6.7% of patients (*n* = 2458) vs. 0.6% of physicians (*n* = 255) and for pain, 1.7% of patients (*n* = 636) vs. 0.5% of physicians (*n* = 207). We found only minor agreements between patients and physicians (maximum Cohen’s kappa: 0.50, 95% confidence interval [0.49;0.51] for depressive mood). Being a female and participation in the randomization were associated with higher odds of reporting an adverse reaction.

**Conclusions:**

In our study, patients’ and physicians’ reports on adverse reactions of acupuncture differed substantially, possibly due to differences in patients’ and physicians’ questionnaires and definitions. For the assessment of safety, we strongly support the inclusion of patients’ and physicians’ reports while ensuring standardization of data collection and definitions.

**Electronic supplementary material:**

The online version of this article (10.1186/s12906-019-2638-x) contains supplementary material, which is available to authorized users.

## Background

Complementary and alternative medicine (CAM) therapies are widely used to treat diseases [1–6]. Acupuncture in particular has been shown to be useful for chronic pain conditions [7–11]. Several studies were performed that evaluated its efficacy, effectiveness, and safety [12–14]. The rate of patient consultations for CAM treatments has been increasing [15]. As a result, the number of reported adverse reactions might increase, especially those which are not always avoidable, such as haematomas, nausea, vomiting, and aggravation of symptoms [13, 16]. Previous observational studies showed that acupuncture can be considered a safe therapy [13, 17, 18], although some case reports might give another impression [12, 19]. However, serious life-threatening adverse reactions of acupuncture, such as pneumothorax, are very rare [12, 13] but have been published in some case reports [20]. Acupuncture treatment for chronic low back and knee pain had been included in routine reimbursements by statutory health insurances in Germany since 2007 [21–24].

Patients who are interested in receiving needle acupuncture treatment should be informed about possible adverse reactions for ethical reasons [12] and patient safety should have a greater priority in acupuncture training [14]. However, the type and frequency of adverse reactions are difficult to compare between the various studies evaluating acupuncture safety [12].

A limitation in many trials is that only health care professionals, especially the physicians, have the responsibility to document (serious) adverse events or adverse reactions [25, 26]. The health care professionals have the professional competence regarding the evaluation of adverse events or adverse reactions, whereas patients have good individual knowledge about their own safety in healthcare [27]. Only a few studies document the adverse reactions by both physician and patients, e.g. [28] with the result that frequency and severity can differ between physicians and patients self-reports.

The aim was to compare patients’ safety reporting with physicians’ safety reporting regarding the safety of acupuncture using data from several large acupuncture trials. Furthermore, associations between patient characteristics and reporting of adverse reactions were evaluated.

## Methods

The present secondary data analysis is based on the Acupuncture in Routine Care (ARC) studies that evaluated the effectiveness of the addition of needle acupuncture treatment [23] compared to usual care only. In those trials, patients and physicians had to complete questionnaires to document safety parameters.

### Study design

The ARC studies were part of the German model project on acupuncture (‘*Modellvorhaben Akupunktur’*) funded by the German statutory health insurances [8, 23, 29]. The project was performed to evaluate the effectiveness of acupuncture treatment in routine medical care, as well as its safety and cost effectiveness [23].

The ARC studies were large pragmatic randomized trials with an additional non-randomized study arm including those patients who refused randomization. The recruitment period was from December 2000 to July 2004, and patients in both acupuncture treatment arms (randomized and non-randomized) received 10–15 sessions of needle acupuncture. Patients were eligible if they were at least 18 years of age and had been suffering from one of the following diseases for more than 6 months: osteoarthritis pain of the knee or hip, low back pain, neck pain, headache, allergic rhinitis/asthma, or dysmenorrhea. For each study, more detailed eligibility criteria were employed [8, 30–34].

### Randomization for needle acupuncture

Of the 50,473 pooled patients over all trials who were asked if they agreed to be randomized, 11,486 agreed and were randomized either to the acupuncture treatment group (ACU, *n* = 5831) or to the control group (CON, *n* = 5655). Participants who did not agree to the randomization were part of the non-randomized acupuncture group (NR-ACU, *n* = 38,987). Participants in the ACU and the NR-ACU group started with the acupuncture treatment immediately, whereas the CON group received acupuncture treatment after 3 months. Needle acupuncture was performed by study physicians with at least 140 h of acupuncture training [8].

### Data collection

At baseline, patient age, gender, school graduation, highest educational degree, occupational status, living situation, diagnosis, health insurance status, and health insurance type were assessed. Data collection regarding safety parameters was performed by questionnaires for patients and their study physicians after a complete treatment cycle.

If either the patient or the physician reported the presence of any side effect caused by acupuncture (adverse reaction) in a short first questionnaire, both received a detailed second questionnaire to report additional information about it, including frequency, duration, time between needle acupuncture and reaction, and treatment need because of adverse reaction. All questions of the questionnaires regarding the safety outcomes in the ARC studies are listed in the supplementary material (see additional file 1).

In the present analysis, only the acupuncture treatment groups ACU and NR-ACU that received the immediate acupuncture (*n* = 44,818) were considered because the CON group received different types of questionnaires regarding the safety parameters.

### Safety parameters

We used the following definitions of the CONSORT statement to differentiate the safety parameters: adverse events are ‘harmful events that occur during a trial.*’*; in contrast, adverse reactions are defined as ‘events for which a causality link to the tested intervention is well established and strong enough (sensitive and specific)’ [35]. Several other institutions in the healthcare sector such as the Food and Drug Administration (FDA), Europeans Medicine Agency (EMA), World Health Organization (WHO), or the German Federal Institute for Drugs and Medical Devices (BfArM) define adverse events and adverse reactions, including drug reactions, in a similar, but not identical way. The definitions are listed in the supplementary material (see additional file 2). In a review by Edwards and Aronson, the differentiation was explained as follows: ‘The terms *adverse effect* and *adverse reaction* are interchangeable’ and ‘must be distinguished from *adverse event*.’ [36].

### Statistical analysis

The primary analysis assessed the agreements for all adverse reactions, which were classified into six categories: i. BLEEDING/HAEMATOMA, ii. INFLAMMATION, iii. PAIN, iv. VEGETATIVE SYMPTOMS, v. NERVE IRRITATION/INJURIES, and vi. OTHERS. The frequencies of the reported adverse reactions are listed, and a description in text form according to the European Commission guidelines is given: very common (≥1/10), common (≥1/100 to < 1/10), uncommon (≥1/1000 to < 1/100), rare (≥1/10,000 to < 1/1000) and very rare (< 1/10,000) [37].

In addition, the agreement between patients’ and physicians’ reports was assessed using Cohen’s kappa (*κ*), a coefficient that measures inter-rater agreement corrected for agreement [38]. Kappa can take values from − 1 to 1 and can be interpreted in accordance with the five levels by Landis and Koch: less than 0.00, poor; 0.00 to 0.20, slight; 0.21 to 0.40, fair; 0.41 to 0.60, moderate; 0.61 to 0.80, substantial; and 0.81 to 1.00, almost perfect agreement [39]. Note that the observed agreement is prevalence-dependent, but the agreement by chance is not.

To assess the association between self-reported adverse reactions by patient or physician (yes/no) and patients’ characteristics, a logistic regression approach was used. Because different participants were treated by the same study physician, the data are clustered. The effect of clustered data was estimated with the intraclass correlation coefficient (ICC) and the design effect (DE) [40]. The ICC estimates the correlation (similarity) of patients’ and physicians’ reports for patients of the same physician based on a null model, which represents a regression model with the variable for clustering only but no further covariates. If the ICC is near 1 and the design effect is much higher than 1, a clustered data structure is present. In the multilevel model developed by Laird and Ware [41], the clustered data structure can be taken into account in the model with physicians as random effects and patient characteristics as fixed effects. In a sensitivity analysis, generalized estimated equation (GEE) models by Liang and Zeger were used [42].

All analyses were performed with the statistics software R (The R Foundation for Statistical Computing, Vienna, Austria, version 3.1.1.) and the packages *lme4* and *geepack* for clustered data regression based on the data set in SPSS format (IBM SPSS Statistics 19). An explorative significance level of 0.05 was used, and multiple test corrections were not applied. Note that the significance of all results (or confidence intervals) should be interpreted only exploratively. Furthermore, it should be noted that for all the following results, missing data were not imputed, and the analyses were based on the respective available data.

## Results

### Patient characteristics

In the ARC studies, *n* = 44,818 patients received immediate acupuncture treatment in the ACU and NR-ACU treatment groups performed by 6727 physicians. Patients who received acupuncture treatment were on average 48.5 ± 14.1 (mean ± standard deviation) years old, and 67.5% were women (Table 1). Of the included patients, 37.1% had at least a high school degree, 59.5% were employees, and 83.8% live in a multi-person household. Characteristics were similar in the ACU and NR-ACU groups. The most common diagnoses for inclusion in the study were headache and neck pain in both groups (ACU: 26.9%, 30.1%; NR-ACU: 29.6%, 26.7%, respectively). On average, approximately seven patients were treated by one study physician (6.6 ± 9.1, median = 4), with a range of only one to more than 50 patients per physician.

**Table 1:** Baseline characteristics for the acupuncture in routine care (ARC) patients by the treatment groups randomized acupuncture (ACU) and non-randomized acupuncture (NR-ACU)

Patient characteristics	ACU *n* = 5831 mean ± sd / *n* (%)	NR-ACU *n* = 38,987 mean ± sd / *n* (%)	Total *n* = 44,818 mean ± sd / *n* (%)
Age in years	48.1 ± 13.8	48.5 ± 14.1	48.5 ± 14.1
Gender			
Male	1883 (32.3)	12,688 (32.5)	14,571 (32.5)
Female	3948 (67.7)	26,299 (67.5)	30,247 (67.5)
Graduation in school (available data *n* = 42,648)			
9 years	1799 (31.9)	11,398 (30.8)	13,197 (30.9)
10 years	1672 (29.6)	10,227 (27.6)	11,899 (27.9)
12/13 years	1945 (34.5)	13,886 (37.5)	15,831 (37.1)
No graduation	47 (0.8)	234 (0.6)	281 (0.7)
Still going to school	23 (0.4)	143 (0.4)	166 (0.4)
Other graduation	154 (2.7)	1120 (3.0)	1274 (3.0)
Highest educational degree (available data *n* = 41,941)			
Apprenticeship	1858 (33.5)	11,612 (31.9)	13,470 (32.1)
Technical school	628 (11.3)	4318 (11.9)	4946 (11.8)
Technical college	981 (17.7)	6175 (17.0)	7156 (17.1)
University of applied sciences	685 (12.3)	4874 (13.4)	5559 (13.3)
University/college	745 (13.4)	5481 (15.1)	6226 (14.8)
No degree	301 (5.4)	1797 (4.9)	2098 (5.0)
Still in apprenticeship or	212 (3.8)	1164 (3.2)	1376 (3.3)
universityOther degree	141 (2.5)	969 (2.7)	1110 (2.6)
Occupational status (available data *n =* 43,909*)*			
Employee	3442 (59.9)	22,687 (59.4)	26,129 (59.5)
Self-employed	239 (4.2)	1905 (5.0)	2144 (4.9)
Unemployed	441 (7.7)	2235 (5.9)	2676 (6.1)
Welfare recipient	17 (0.3)	134 (0.4)	151 (0.3)
Student	123 (2.1)	901 (2.4)	1024 (2.3)
Pensioner	1295 (22.5)	8940 (23.4)	10,235 (23.3)
Others	189 (3.3)	1361 (3.6)	1550 (3.5)
Living situation (available data *n* = 42,615)			
Multiperson household	4739 (84.1)	30,960 (83.7)	35,699 (83.8)
Single person household	898 (15.9)	6018 (16.3)	6916 (16.2)
Diagnosis			
Headache	1571 (26.9)	11,545 (29.6)	13,116 (29.3)
Asthma/allergic rhinitis	671 (11.5)	5342 (13.7)	6013 (13.4)
Low back pain	1449 (24.8)	8532 (21.9)	9981 (22.3)
Neck pain	1753 (30.1)	10,392 (26.7)	12,145 (27.1)
Dysmenorrhea	101 (1.7)	448 (1.1)	549 (1.2)
Arthritis	286 (4.9)	2728 (7.0)	3014 (6.7)
Health insurance status (available data *n* = 43,909)			
Member	4447 (77.4)	29,199 (76.5)	33,646 (76.6)
Spouse	1202 (20.9)	8256 (21.6)	9458 (21.5)
Child	97 (1.7)	708 (1.9)	805 (1.8)
Health insurance type (available data *n* = 43,909)			
Mandatory insured	3644 (63.4)	22,598 (59.2)	26,242 (59.8)
Voluntary insured	2102 (36.6)	15,565 (40.8)	17,667 (40.2)

### Comparing patient and physician reports

**Fig. 1:**
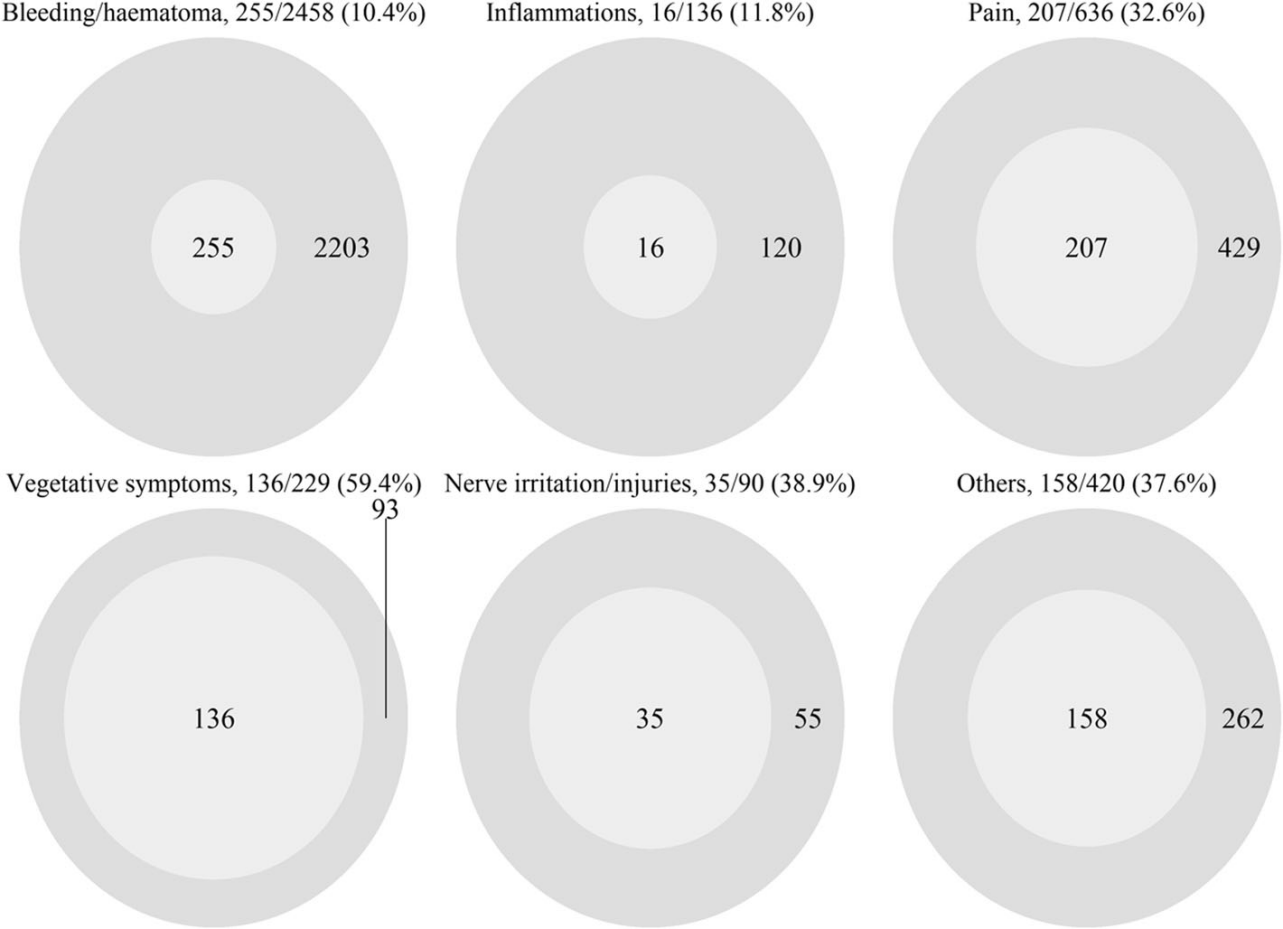
Physicians (light grey inner circle) to patients (outer circle) reported adverse reactions in six categories: bleeding/haematoma (*n* = 255 physicians and *n* = 2458 patients reports, the ratio represents 10.4%), inflammation, pain, vegetative symptoms, nerve irritation/injuries, and others (for numbers see Table 2)

The comparison of patient- and physician-reported adverse reactions during the trial is provided as absolute frequencies, proportions (%), and categories (Table 2). It shows differences between patients’ and physicians’ ratings for the main categories: BLEEDING/HAEMATOMA (patients: 2458 (6.7%), considered as ‘common’ vs. physicians: 255 (0.6%), ‘uncommon’), PAIN (636 (1.7%), ‘common’ vs. 207 (0.5%), ‘uncommon’), INFLAMMATION (136 (0.4%), ‘uncommon’ vs. 16 (0.04%), ‘rare’, NERVE IRRITATION/INJURIES (90 (0.2%), ‘uncommon’ vs. 35 (0.1%), ‘rare’), and OTHERS (420 (1.1%), ‘common’ vs. 158 (0.4%), ‘uncommon’). However, VEGETATIVE SYMPTOMS was reported in the same frequency category by patients (229 (0.6%), ‘uncommon’) and physicians (136 (0.3%), ‘uncommon’). The proportions of physicians’ to patients’ reports for the adverse reaction categories are illustrated (Fig. 1).

**Table 2:** Frequency of reported adverse reactions by patients and physicians sorted by categories in frequencies and proportions, description in text form, and agreement as Cohen’s kappa (**κ**) coefficient with 95% confidence interval (CI) for 36,792 and 42,811 available data of 44,818 patients

	Frequency *n* (%)		Description^a^		Cohen’s kappa (*κ*)^d^	
	Patient reported *available data n = 36,792*	Physician reported *available data n = 42,811*	Patient reported *available data n = 36,792*	Physician reported *available data n = 42,811*	Kappa	95% CI
Bleeding/haematoma	2458 (6.681)	255 (0.596)	common	uncommon	0.11	0.10–0.11
Inflammation	136 (0.370)	16 (0.037)	uncommon	rare		
Inflammation	129 (0.351)	13 (0.030)	uncommon	rare	0.09	0.08–0.10
Local infection	7 (0.019)	3 (0.007)	rare	very rare	0.00	−0.01 − 0.01
Pain	636 (1.729)	207 (0.484)	common	uncommon		
Other pain	215 (0.584)	38 (0.090)	uncommon	rare	0.08	0.07–0.09
Headache	197 (0.535)	49 (0.114)	uncommon	uncommon	**0.21**	0.20–0.21
Aggravation of symptoms	89 (0.242)	65 (0.152)	uncommon	uncommon	0.09	0.08–0.10
Local muscle pain	73 (0.198)	21 (0.049)	uncommon	rare	0.02	0.01–0.03
Strong pain during needling	61 (0.166)	33 (0.077)	uncommon	rare	0.02	0.01–0.03
Generalized muscle pain	1 (0.003)	1 (0.002)	very rare	very rare	0.00	−0.01 − 0.01
Vegetative symptoms	229 (0.622)	136 (0.320)	uncommon	uncommon		
Vertigo	78 (0.212)	31 (0.072)	uncommon	rare	0.16	0.15–0.17
Other cardiovascular disturbance	62 (0.169)	62 (0.145)	uncommon	uncommon	0.16	0.15–0.17
Nausea	48 (0.130)	17 (0.040)	uncommon	rare	**0.21**	0.20–0.22
Unconsciousness	10 (0.027)	8 (0.019)	rare	rare	**0.27**	0.26–0.28
Breathing difficulties	9 (0.024)	6 (0.014)	rare	rare	0.00	−0.01 − 0.01
Sweating	9 (0.024)	5 (0.012)	rare	rare	0.18	0.17–0.19
Tachycardia	8 (0.022)	6 (0.014)	rare	rare	0.18	0.17–0.19
Increase in blood pressure	2 (0.005)	1 (0.002)	very rare	very rare	0.00	−0.01 − 0.01
Palpitations^b^	1 (0.003)	0 (0.000)	very rare	–	0	–
Constipation^b^	1 (0.003)	0 (0.000)	very rare	–	0	–
Enterospasm^b^	1 (0.003)	0 (0.000)	very rare	–	0	–
Nerve irritation/injuries	90 (0.245)	35 (0.082)	uncommon	rare		
Hypaesthesia	44 (0.120)	7 (0.016)	uncommon	rare	0.04	0.03–0.05
Nerve irritations	24 (0.065)	4 (0.009)	rare	very rare	0.08	0.07–0.08
Paraesthesia	12 (0.033)	22 (0.051)	rare	rare	0.06	0.05–0.07
Nerve injury	5 (0.013)	2 (0.005)	rare	very rare	0.00	−0.01 − 0.01
Paresis ^b^	5 (0.013)	0 (0.000)	rare	–	0	–
Others	420 (1.142)	158 (0.369)	common	uncommon		
Fatigue	80 (0.217)	16 (0.037)	uncommon	rare	0.16	0.15–0.17
Swelling	70 (0.190)	11 (0.026)	uncommon	rare	0.12	0.11–0.12
Other dermal phenomena	36 (0.098)	15 (0.035)	rare	rare	**0.26**	0.25–0.27
Other neurological complaints	27 (0.073)	1 (0.002)	rare	very rare	0.00	0.00–0.00
Itching	24 (0.065)	3 (0.007)	rare	very rare	0.09	0.08–0.09
Worsening health state	23 (0.063)	10 (0.023)	rare	rare	0.07	0.06–0.08
Redness	21 (0.057)	16 (0.037)	rare	rare	0.06	0.05–0.07
Collapse	18 (0.049)	17 (0.040)	rare	rare	0.19	0.18–0.20
Restricted movements	15 (0.041)	1 (0.002)	rare	very rare	0.00	−0.01 − 0.01
Other mood swings	13 (0.035)	8 (0.019)	rare	rare	0.11	0.09–0.12
Tinnitus	10 (0.027)	4 (0.009)	rare	very rare	**0.29**	0.28–0.30
Vomiting	9 (0.024)	4 (0.009)	rare	very rare	0.17	0.16–0.18
Feeling of coldness	8 (0.022)	3 (0.007)	rare	very rare	0.00	−0.01 − 0.01
Menstrual problems	8 (0.022)	3 (0.007)	rare	very rare	**0.25**	0.24–0.26
Anxiety	8 (0.022)	11 (0.030)	rare	rare	**0.35**	0.34–0.36
Sleep disturbance	6 (0.016)	2 (0.005)	rare	very rare	0.00	−0.01 − 0.01
Disturbed vision	6 (0.016)	2 (0.005)	rare	very rare	0.00	-0-01 − 0.01
Diarrhoea	5 (0.014)	3 (0.007)	rare	very rare	**0.29**	0.28–0.30
Needle forgotten	4 (0.011)	1 (0.002)	rare	very rare	0.00	−0.01-0.01
Imbalance	4 (0.011)	1 (0.002)	rare	very rare	0.00	-0.01-0.01
Other gastrointestinal complaints	4 (0.011)	3 (0.007)	rare	very rare	0.00	-0.01-0.01
Joint problems	3 (0.008)	3 (0.007)	very rare	very rare	0.00	-0.01-0.01
Depressive mood	3 (0.008)	2 (0.005)	very rare	very rare	**0.50**	0.49–0.51
Eye irritation	3 (0.008)	1 (0.002)	very rare	very rare	0.00	-0.01-0.01
Burns after moxibustion	2 (0.005)	6 (0.014)	very rare	rare	0.00	-0.01-0.01
Systemic infection	2 (0.005)	4 (0.009)	very rare	very rare	0.00	-0.01-0.01
Poor concentration^b^	2 (0.005)	0 (0.000)	very rare	–	0	–
Gastrospasm	2 (0.005)	1 (0.002)	very rare	very rare	0.00	-0.01-0.01
Vascular injuries^b^	1 (0.003)	0 (0.000)	very rare	–	0	–
Nightmares^b^	1 (0.003)	0 (0.000)	very rare	–	0	–
Restlessness/nervousness	1 (0.003)	3 (0.007)	very rare	very rare	0.00	-0.01-0.01
Shivering^b^	1 (0.003)	0 (0.000)	very rare	–	0	–
Pneumothorax^c^	0 (0.000)	1 (0.000)	–	very rare	0	–
Other organ injuries^c^	0 (0.000)	1 (0.000)	–	very rare	0	–
Disorientation^c^	0 (0.000)	1 (0.000)	–	very rare	0	–
**Average kappa (** ***κ*** **)**					**0.21**	**0.20–0.22**

Note: a kappa coefficient can only be calculated if both the patient and physician reported a reaction; kappa values above 0.2 indicate fair agreement and are marked in bold

^a^Description of the frequencies in text form according to the guideline of the European Commission to describe the frequencies of adverse effects or reactions of medical products: very common: (≥1/10), common (≥1/100 to < 1/10), uncommon (≥1/1000 to < 1/100), rare (≥1/10,000 to < 1/1000) and very rare (< 1/10,000) [37].

^b^Reported only by patient

^c^Reported only by physician

^d^Interpretation of Cohen’s kappa (*κ*): < 0, poor; 0–0.20, slight; 0.21–0.40, fair; 0.41–0.60, moderate; 0.61–0.80, substantial; 0.81–1.00, almost perfect; 1, perfect agreement

Based on available data, 79% (*n* = 696) of the patients stated that they had informed their physician about their adverse reaction, whereas only 25% (*n* = 426) of the physicians reported they had learned this from their patients. Most of the physicians (88.5%, *n* = 1512) had not observed the adverse reactions themselves.

The agreements between patient- and physician-reported adverse reactions as measured by Cohen’s kappa differed for the various categories (Table 2). Depressive mood had the highest kappa value of 0.50, which represents a moderate agreement between patient and physician. Anxiety (Cohen’s kappa 0.35), tinnitus (0.29), and diarrhoea (0.29) also showed higher values. Many kappa values, however, represented only slight agreement with kappa values between 0.0 and 0.2 (not unexpected due to small prevalence). For several adverse reactions, the kappa value was estimated as agreement by chance (kappa = 0), e.g., for local infection, generalized muscle pain, increase in blood pressure, nerve injury, even though these adverse reactions had above-average prevalence. Specific questions on serious adverse events were included in the physicians’ questionnaires (see additional file 1), but frequencies were too low to be analysed meaningfully.

### Association between reported adverse reactions and baseline characteristics

The ICC, estimating the similarity of patients’ and physicians’ reports for patients of the same physician, was 0.12 based on patients’ reports and 0.90 for physicians’ reports. Therefore, 12 and 90% of the total variability is between the patients treated by the same study physicians, and this effect will be considered in the subsequent logistic regression analyses.

To assess associations between patients’ characteristics and patient-reported or physician-reported adverse reactions (yes/no), a multivariable multilevel logistic regression was applied. Female patients showed higher odds of reporting adverse reactions than males (OR 1.96, 95% CI [1.76;2.17], Table 3). Patients who had agreed to be randomized showed higher odds of reporting (1.24 [1.11;1.39]) than patients who had not agreed to randomization. Older patients (for a 10-year increase in age) reported significantly less adverse reactions (0.82 [0.82;0.90]). Patients with a higher educational degree were more likely to report adverse reactions (1.39 [1.22;1.59] for 12/13 years in school; 1.16 [1.05;1.29] for academic degree in college/university) than patients with a lower degree.

**Table 3:** Association between patient characteristics and reporting of adverse reaction (yes/no) from patients and physicians (multivariable multilevel logistic regression, yielding adjusted* odds ratios and 95% confidence intervals (CI))

Patient characteristics	Patient report adverse reaction *available data n = 36,792* *(yes/no: 3368/33,424)*		Physician report adverse reaction *available data n = 41,822* *(yes/no: 651/41,171)*	
	Odds ratio	95% CI	Odds ratio	95% CI
Age (10 year increase)	0.82	0.82–0.90	1.00	0.90–1.10
Gender				
Female (vs. male)	1.96	1.76–2.17	2.39	1.87–3.15
Graduation in school years (reference: 9 years)				
12/13 years	1.39	1.22–1.59	2.32	1.65–3.26
10 years	1.26	1.12–1.42	1.17	0.87–1.59
Other or no graduation	1.18	0.94–1.48	1.15	0.64–2.08
Highest degree (reference: apprenticeship)				
College/university	1.16	1.04–1.29	1.03	0.79–1.33
Technical school/college	1.15	1.01–1.31	0.57	0.41–0.79
Other or no degree	0.90	0.77–1.05	0.98	0.68–1.41
Occupational status (reference: employed)				
Unemployed or welfare-recipient	1.21	1.03–1.41	0.42	0.25–0.71
Student or other	1.19	1.01–1.40	1.53	1.06–2.21
Pensioner	1.05	0.91–1.21	0.66	0.46–0.96
Living situation				
Multiperson household (vs. single person)	0.93	0.83–1.03	1.12	0.85–1.47
Randomization				
Randomized (vs. non-randomized)	1.24	1.11–1.39	2.10	1.59–2.78
Diagnosis (reference: headache)				
Asthma/allergic rhinitis	1.03	0.91–1.16	0.97	0.70–1.33
Low back pain	0.85	0.76–0.96	0.69	0.51–0.95
Neck pain	1.01	0.91–1.12	1.09	0.83–1-41
Dysmenorrhea	0.86	0.62–1.19	0.71	0.29–1.71
Arthritis	0.85	0.67–1.07	0.40	0.21–0.76
Health insurance status (reference: member)				
Spouse	1.00	0.90–1.13	0.90	0.67–1.20
Child	1.14	0.83–1.57	0.61	0.25–1.50
Health insurance type				
Voluntary insured (vs. mandatory)	0.93	0.85–1.02	0.83	0.65–1.05

*Adjusted for all other factors listed

For physicians, the tendencies for associations are similar, but the ORs are less precise (Table 3). Study physicians reported significantly more adverse reactions for female than for male patients (2.39 [1.87;3.15]), for patients with higher degrees (2.32 [1.65;3.26] for 12/13 years in school), and for patients who had agreed to be randomized before the studies (2.10 [1.59;2.78]). The differences between the two statistical approaches, multilevel model and GEE models, are negligible (GEE model results not shown).

## Discussion

We evaluated the reporting of adverse reactions in a secondary data analysis of a large semi-randomized controlled clinical trial on acupuncture for chronic pain patients. We compared patients’ and physicians’ reports regarding the frequency of adverse reactions and evaluated their agreement. Overall, the patients reported on average three times more adverse reactions than their physicians. The most commonly reported adverse reaction was bleeding/haematoma for both patients and physicians, similar to a study by Witt et al. [13]. Despite this, many types of adverse reactions were seldom reported, especially life-threatening adverse reactions such as pneumothorax [13, 20, 43]. No or only slight chance-corrected agreements existed. However, differences in actual frequency did not necessarily result in differences regarding frequency category commonly used in product descriptions [37]. Moreover, we observed that the chance of reporting an adverse reaction either by the patient or the physician was higher for patients who had agreed to be randomized at baseline, i.e., who were willing to participate in an RCT, were female, and had a higher education degree. Various reasons might explain the difference between patients’ and physicians’ reporting. In general, the physician is equipped with more medical knowledge than the patient due to long-term medical training and professional experience, which can impact the reporting of adverse reactions, especially when the causality is vague. Indeed, it is feasible that the patient is best positioned to report his or her own symptoms [44].

The communication about the treatment and about its potential adverse reactions, the motivation and time for reporting, the disease treated, and the general educational background might also impact reporting. Furthermore, the method of documenting adverse reactions might have an impact. In our study, the patient’s question used to specify adverse reactions offered tick boxes for bleeding/haematoma and local inflammation as examples, whereas the physician’s included a free text answer to this question. This and the fact that these reactions might not be considered side effects by acupuncturists from the perspective of traditional Chinese medicine have possibly contributed to the differences in these categories. Although we included a comparably large number of cases and treatments with more than 44,000 chronic pain patients, conclusions regarding the specific frequency of adverse reactions in acupuncture should be drawn very carefully when they are only based on our present study. For the evaluation of acupuncture safety, other even larger studies were specifically designed, performed and published [13, 17, 45].

This study has some limitations. Firstly, we used secondary data from December 2000 to July 2004 which were not primarily designed to evaluate the differences between physicians’ and patients’ reporting of adverse reactions. The primary aim of the ARC trials was the evaluation of efficacy, while evaluating safety was only one of many secondary outcomes. We did not adjust for multiple comparisons.

A further problem is that the patients and physicians do not rate completely independent because many adverse reactions are invisible to the study physician and have to be reported by the patient to the physician first, and the physician might explain the definition of adverse reactions to the patient. Hence, physician’s reports based on the patient’s reports could validate the patient’s report, assuming the physician’s assessment can serve as the gold standard. However, this might cause under-reporting, whereas over-reporting of too many unjustified adverse reactions could cause difficulties when explaining the safety characteristics of the intervention in a real life setting. A further limitation of our study is that the assessment of adverse reactions was based on retrospective self-reports, which can be influenced by recall bias [46]. The lack of differentiation between adverse events and adverse reactions caused by acupuncture could be an additional reason for the differential reporting. Definitions according to WHO, FDA or EMA differ by nuance [35, 47–49]. In the literature, these terms are sometimes used synonymously (e.g., [50, 51]). The exact definitions of reputable institutions are listed in the supplemental material (see additional file 2).

In the physicians’ questionnaire in this study, we included a definition for an adverse reaction that referred to its noxious and unintended character to separate it from an adverse event. In contrast, the patient’s questionnaire did not include any explanation to improve clarity and usability of the questionnaire and because only adverse reactions and not adverse events had to be reported by patients. The difference in the questionnaires may to some extent explain differences in reporting of some adverse reactions, such as bleeding/haematoma that is sometimes intended by acupuncturists or pain, but not the differences for adverse reactions such as vertigo or fatigue. For future studies, we recommend a similar application of written definitions for both the physician and the patient questionnaire. However, tick boxes or free text should also be applied in a similar way. Not only who assesses but also how the assessment is performed can cause large differences in reported rates as shown in an RCT by Bent et al. [52]. This study compared three methods (1. an open-ended question, 2. an open-ended, defined question, and 3. a checklist of 53 common side effects) to assess adverse events experienced by study participants. The percentage of patients reporting any adverse events was much higher in the group using the checklist (77%) than in the first (14%) or second group (13%). This demonstrates the complexity of reporting and standards.

Strikingly, most of the studies on the safety characteristics of acupuncture are either based on therapists’/physicians’ or patients’ reporting but not of both [53–56]. Fromme et al. investigated the clinician reporting of adverse reactions during chemotherapy [57]. In the study, 37 men with prostate cancer reported their adverse events, and the agreements with the study physicians using Cohen’s kappa was determined. The total Cohen’s kappa value was 0.15, which represents slight agreement, and was similar to our results. For rheumatoid arthritis, the reporting of adverse drug events between patients (*n* = 4246) and physicians differed; even for serious adverse events, the agreement was only 37% [58], whereas patients reported more events, which is similar to our results.

In a study comparing adverse events reported in post-discharge patient interviews with adverse events detected by medical record review, the agreement for adverse events (kappa = 0.20) and serious adverse events (kappa = 0.33) was low and comparable to our agreement results [59]. In contrast, in an oncology study in 2005, the agreement of 400 patients with their clinicians was higher (kappa up to 0.5) [60]. Especially, for observable reactions, the agreement was higher than for subjective ones [60].

A standardized reporting and documentation of both adverse events and reactions is essential [35, 61, 62]. For drug safety, the FDA developed a reporting system in 1998 [63]. For non-interventional pain studies, there are guidelines as CONSORT or ACTTION to document adverse reactions, but even these guidelines do not provide a high degree of detail [64]. In oncology, there are currently some documentation tools to combine the analytical and professional side of physicians and individual patient’s side using quality of life, symptoms, and patient-reported outcomes to enhance patient-clinician communication and to enable early detection of toxicities [60, 65]. In a British acupuncture study, the adverse events were monitored in a standardized way with self-reports by patient at each acupuncture session [66]. However, the documentation of safety by patient reports is still not standardized in clinical trials and health care. Even for the obligatory adverse drug reaction documentation, various information systems are used [67]. Standardized electronic web-based documentation software or intuitive mobile apps in contrast to classical methods (phone, questionnaire) could support the complete and harmonized documentation of adverse reactions [67, 68]. Further, it is important to differentiate between adverse events and adverse reactions and to evaluate a possible causal link to the intervention.

We think that both patients’ and physicians’ reports should be included when evaluating safety aspects of a medical intervention while electronic documentation tools might support this. Patients (or their relatives) can play an important role in signalling safety aspects in clinical trials as well as in routine care [62, 69] and can help the patient-centred approach in the future.

Regular communication between the physicians, other clinical staff and patients and the standardization of documents, including clarification of definitions, might help to minimize differences.

## Conclusions

In our study, patients’ and physicians’ reports of adverse reactions of acupuncture differed substantially, possibly due to differences in patients’ and physicians’ questionnaires and definitions. The use of frequency categories has been shown to be useful and able to compensate for reporting differences. For the assessment of safety parameters, we strongly support the inclusion of both patients’ and physicians’ reports while ensuring standardization of data collection and definitions.

## Additional files

Additional file 1: Questions about safety outcomes for patients and physicians during the Acupuncture in Routine Care study (ARC), shown are German wordings and their English translations. (DOCX 21 kb)

Additional file 2: Definitions of the terms adverse event (AE), adverse reaction (AR) and suspected adverse reaction (SAR) by international institutions. (DOCX 20 kb)

## Data Availability

The datasets generated and/or analysed during the current study are not publicly available due to patient confidentiality and informed consent cannot be given by deceased. Information are available from the corresponding author on reasonable request.

## Abbreviations

ACU: Randomized acupuncture group
ARC: Acupuncture in Routine Care
BfArM: German Federal Institute for Drugs and Medical Devices
CAM: Complementary and alternative medicine
CI: Confidence interval
CON: Control group
DE: Design effect
EMA: Europeans Medicine Agency
FDA: Food and Drug Administration
GEE: Generalized estimated equation
ICC: Intraclass correlation coefficient
NR-ACU: Non-randomized acupuncture group
OR: Odds Ratio
WHO: World Health Organization
