# Supplementary material for: Comparing physicians’ and patients’ reporting on adverse reactions in randomized trials on acupuncture—a secondary data analysis
Source: BMC Complement Altern Med. 2019 Aug 22;19:223. doi: 10.1186/s12906-019-2638-x (PMC6704486; doi:10.1186/s12906-019-2638-x)
Supplement: Supplementary file 1 — Questions about safety outcomes for patients and physicians during the Acupuncture in Routine Care study (ARC), shown are German wordings and their English translations. (DOCX 21 kb) [file 12906_2019_2638_MOESM1_ESM.docx]

SUPPLEMENTARY MATERIAL

Table A.1 *Questions about safety outcomes for patients and physicians during the Acupuncture in Routine Care study (ARC), shown are German wordings and their English translations.*

| Final patient questionnaire  Did you experience any side effect caused by acupuncture treatment (🞏yes/🞏no)?  If yes, which ones (bleeding/haematoma at the puncture site, inflammation at the puncture site, others (please specify))?  How were these side effects treated (doing nothing, I have treated myself, because of side effects additional treatment with the same doctor)? | Patientenabschlussbogen  Sind bei Ihnen Nebenwirkungen durch die Akupunkturbehandlung aufgetreten (🞏Ja/🞏Nein)?  Wenn ja, welche (Blutung/Bluterguss an der Einstichstelle, Entzündung an der Einstichstelle, Sonstiges (bitte eintragen))?  Wie wurden diese Nebenwirkungen behandelt (gar nicht, habe ich selbst behandelt, wegen Nebenwirkungen zusätzliche Behandlung bei dem gleichen Arzt)? |
| --- | --- |
| Final physician questionnaire  Did side effects of acupuncture occur (🞏yes/🞏no)? This refers to all side effects which, from your point of view, were caused by the acupuncture treatment. | **Arztabschlussbogen**  Sind durch die Akupunktur hervorgerufene Nebenwirkungen aufgetreten (🞏Ja/🞏Nein)? Gemeint sind alle Nebenwirkungen, die aus Ihrer Sicht ursächlich mit der Akupunkturbehandlung in Verbindung stehen. |
| Patient questionnaire on side effects of acupuncture  What side effect did you experience? Please name the most serious one first and fill in the questionnaire for this side effect. All other side effects should be mentioned by name only.  When did the side effect occur (🞏during/🞏after the acupuncture session)?  If the side effect occurred after acupuncture, how long after treatment did you notice it (time in minutes/ hours/ days)?  How often did the side effect occur (🞏once/ 🞏several times)? If several times, how often?  At which or after which acupuncture session did the side effect occur? If this has occurred during or after several sessions, please list several crosses.  How long did the side effect last on average (time in minutes, hours, or days)?   Was the side effect related to any pain (🞏yes/🞏no)?  How strong do you rate the pain on an eleven-point scale (from 0=no pain to 10=intolerable, permanent pain)?  Have you told your doctor about the side effect (🞏yes/🞏no/🞏don’t know)?  *In addition, patients were also asked about the effect of acupuncture treatment.* | **Patienten-Fragebogen zu Nebenwirkungen bei Akupunktur**  Welche Nebenwirkung ist bei Ihnen aufgetreten? Bitte nennen Sie zuerst die schwerwiegendste und füllen Sie bezogen auf diese Nebenwirkung den Fragebogen aus. Alle weiteren Nebenwirkungen nennen Sie bitte nur namentlich.  Zu welchem Zeitpunkt ist die Nebenwirkung aufgetreten (🞏während/🞏nach der Akupunktur)?  Wenn die Nebenwirkung nach der Akupunktur aufgetreten ist, in welchem zeitlichen Abstand zur Behandlung haben Sie sie zuerst bemerkt (Zeitangabe in Minuten/ Stunden/ Tagen)?  Wie oft ist die Nebenwirkung aufgetreten? (**🞏**einmal/ **🞏**mehrmals) Falls mehrmals, wie oft?  Bei oder nach der wievielten Akupunktursitzung ist die Nebenwirkung aufgetreten? Wenn diese bei oder nach mehreren Sitzungen aufgetreten ist, verzeichnen Sie bitte mehrere Kreuze.  Wie lange hat die Nebenwirkung durchschnittlich angehalten (Zeitangabe in Minuten/ Stunden/ Tagen)?  War die Nebenwirkung mit Schmerzen verbunden (🞏Ja/🞏Nein)?  Wie stark beurteilen Sie die Schmerzen auf einer 11stufigen Skala (0=keine Schmerzen, 10=unerträgliche, dauerhafte Schmerzen)?  Haben Sie dem Arzt die Nebenwirkung mitgeteilt (🞏Ja/🞏Nein/🞏weiß nicht)?  *Die Patienten wurden zusätzlich zum Effekt der Akupunkturbehandlung gefragt.* |
| Doctor's questionnaire on side effects of acupuncture  Are you aware of serious adverse events (SAE) during the period of acupuncture treatment (🞏yes/🞏no)? If yes, which SAEs?  (SAE are those which are fatal or life-threatening, lead to permanent damage, in-patient treatment, or extension of the in-patient stay. Congenital malformation or the occurrence of a malignant tumor are regarded as SAE in any case.)    Were the SAE related to the acupuncture treatment (🞏yes/🞏no)?  Are you aware of side effects of the acupuncture treatment?  (Side effect: A reaction, which is harmful and unintended and which occurs at a dosage normally used in humans for the prevention, diagnosis or therapy of diseases, or changes of physiological functions.)  How confident are you about the relation with acupuncture (🞏certain/ 🞏moderate/ 🞏uncertain)?   Please specify the most serious side effect first and complete the questionnaire based on this side effect.  How did you know about the side effect (🞏of the patient, 🞏 I observed it myself)?  When did the side effect occur (🞏during/ 🞏after the acupuncture session)?  If the side effect occurred after acupuncture, how long after treatment did you observe it (time in minutes/hours/ days)?  How often did the side effect occur (🞏once/ 🞏several times)? If several times, how often?  How long did the side effect last on average (time in minutes, hours, or days)?  Was the side effect related to any pain (🞏yes/🞏no)? | **Arzt-Fragebogen zu Nebenwirkungen bei Akupunktur**  Sind Ihnen schwerwiegende unerwünschte Ereignisse (SUE) im Zeitraum der Akupunkturbehandlung bekannt (🞏Ja/🞏Nein)? Wenn ja, welche SUEs?  (SUE sind solche, die tödlich oder lebensbedrohlich sind, zu bleibenden Schäden führen oder eine stationäre Behandlung oder  Verlängerung des stationären Aufenthaltes erforderlich machen. Angeborene Fehlbildung oder das Auftreten eines bösartigen Tumors werden in jedem Fall als SUE angesehen.)  Standen die SUEs im Zusammenhang mit der Akupunktur (🞏Ja/🞏Nein)?  Sind Ihnen Nebenwirkungen der Akupunktur bekannt?  (Nebenwirkung: eine Reaktion, die schädlich und unbeabsichtigt ist und welche bei Dosen auftritt, die üblicherweise beim Menschen für die Prophylaxe, Diagnose oder Therapie von Krankheiten oder die Veränderungen physiologischer Funktionen angewendet werden.)  Wie gesichert scheint Ihnen der Zusammenhang mit der Akupunktur (🞏sicher/ 🞏mittel/ 🞏unsicher)?   Bitte nennen Sie zuerst die schwerwiegendste Nebenwirkung und füllen Sie bezogen auf diese Nebenwirkung den Fragebogen aus.  Wie haben Sie von der Nebenwirkung erfahren (🞏 vom Patienten, 🞏 habe ich selbst beobachtet)?  Zu welchem Zeitpunkt ist die Nebenwirkung bei dem Patienten aufgetreten (🞏während/ 🞏nach der Akupunktur)?  Wenn die Nebenwirkung nach der Akupunktur aufgetreten ist, in welchem zeitlichen Abstand zur Behandlung wurde sie zuerst beobachtet (Zeitangabe in Minuten/ Stunden/ Tagen)?  Wie oft ist die Nebenwirkung aufgetreten (🞏einmal/ 🞏mehrmals)? Falls mehrmals, wie oft?  Wie lange hat die Nebenwirkung durchschnittlich angehalten (Zeitangabe in Minuten/ Stunden/ Tagen)?  War die Nebenwirkung mit Schmerzen verbunden (🞏Ja/🞏Nein)? |
