# Supplementary material for: Comparing physicians’ and patients’ reporting on adverse reactions in randomized trials on acupuncture—a secondary data analysis
Source: BMC Complement Altern Med. 2019 Aug 22;19:223. doi: 10.1186/s12906-019-2638-x (PMC6704486; doi:10.1186/s12906-019-2638-x)
Supplement: Supplementary file 2 — Definitions of the terms adverse event (AE), adverse reaction (AR) and suspected adverse reaction (SAR) by international institutions. (DOCX 20 kb) [file 12906_2019_2638_MOESM2_ESM.docx]

SUPPLEMENTARY MATERIAL

Table A.2 *Definitions of the terms adverse event (AE), adverse reaction (AR) and suspected adverse reaction (SAR) by international institutions.*

| Term | Institution | Connection to Drugs | Definition and reference |
| --- | --- | --- | --- |
| AE | CONSORT | No | Harmful events that occur during a trial.^27^ |
| AE | EMA | No | Any untoward medical occurrence in a patient or clinical trial subject administered a medicinal product and which does not necessarily have a causal relationship with this treatment. An adverse event can therefore be any unfavourable and unintended sign, symptom, or disease temporally associated with the use of a medicinal product, whether or not considered related to the medicinal product.^41^ |
| AE | FDA | Yes | Any untoward medical occurrence associated with the use of a drug in humans, whether or not considered drug related.^39^ |
| AE | WHO | No | Any untoward medical occurrence that may appear during treatment with a pharmaceutical product but which does not necessarily have a causal relationship with the treatment. |
| AR | CONSORT | No | Events for which a causality link to the tested intervention is well established and strong enough (sensitive and specific).^27^ |
| AR | EMA | No | A response to a medicinal product which is noxious and unintended. Response in this context means that a causal relationship between a medicinal product and an adverse event is at least a reasonable possibility.^41^ |
| AR | FDA |  | Not applicable |
| AR | WHO, BfArM | No | A response to a drug which is noxious and unintended, and which occurs at doses normally used in man for the prophylaxis, diagnosis, or therapy of disease, or for the modifications of physiological function.^40^ |
| SAR | CONSORT |  | Not applicable |
| SAR | EMA | No | One or more suspected AR. The report does not also qualify as a valid Individual case report safety [ICSR] if it is reported that the patient experienced an unspecified AR and there is no information provided on the type of AR experienced.^41^ |
| SAR | FDA | Yes | Suspected adverse reaction means any adverse event for which there is a reasonable possibility that the drug caused the adverse event. For the purposes of IND safety reporting, "reasonable possibility" means there is evidence to suggest a causal relationship between the drug and the adverse event. Suspected adverse reaction implies a lesser degree of certainty about causality than adverse reaction, which means any adverse event caused by a drug.^39^ |
